# Impact of different endocrine therapies on bone mineral density and fracture risk in postmenopausal breast cancer patients: a meta-analysis

**DOI:** 10.3389/fmed.2025.1665389

**Published:** 2025-12-01

**Authors:** Haoran Qu, Juntao Wang, Zhekun Wang, Bingjie Guan, Yuling Zheng

**Affiliations:** 1 Department of Hematology and Oncology, The First Affiliated Hospital of Henan University of Chinese Medicine, Zhengzhou, China; 2 Department of Oncology, The Third Affiliated Hospital of Henan University of Chinese Medicine, Zhengzhou, China

**Keywords:** postmenopausal breast cancer, endocrine therapy, bone mineral density, fracture risk, systematic review, meta-analysis

## Abstract

**Objective:**

To investigate the effects of different endocrine therapies (tamoxifen, aromatase inhibitors, selective estrogen receptor modulators) on bone mineral density (BMD) and fracture risk in postmenopausal breast cancer patients.

**Methods:**

A systematic search was conducted in PubMed, Medline, Web of Science, Cochrane Library, and EMBASE databases to identify randomized controlled trials (RCTs). The quality of RCTs was assessed using the Cochrane Risk of Bias Tool, Meta-analysis was performed using RevMan 5.3 software, with primary outcome measures including changes in BMD and fracture risk.

**Results:**

A total of five studies involving 20,531 patients were included. Meta-analysis results showed: Osteoporosis incidence: Pooled analysis of two studies yielded pooled odds ratio OR of 0.35 (95% CI: 0.04, 3.00), indicating a slight but non-significant advantage in reducing fracture risk in the experimental group compared to the control group (*Z* = 0.96, *P* = 0.34). This finding is based on only two studies, so there is uncertainty associated with it. Heterogeneity was low (I^2^ = 97%). fracture risk: The pooled OR from four studies was 1.40 (95% CI: 1.25, 1.57), demonstrating a statistically significant increase in fracture risk in the experimental group (*Z* = 5.92, *P* < 0.001) with low heterogeneity (*I*^2^ = 29%).

**Conclusion:**

The conclusions of this analysis were constrained by the limited number of available studies. Different endocrine therapies might exert varying effects on bone health. Aromatase inhibitors significantly reduce BMD and increase fracture risk, whereas selective estrogen receptor modulators might have a protective effect on bone. These relationships need confirmation in larger studies. Clinicians should consider bone health among other factors when selecting endocrine therapy.

## Introduction

Breast cancer remains one of the most prevalent malignancies among women, and endocrine therapy plays a pivotal role in the management of postmenopausal breast cancer patient (1, 2). With the prolonged survival of breast cancer patients, increasing attention has been directed toward bone health-related issues associated with endocrine therapy (3). Postmenopausal women inherently experience accelerated bone loss due to declining estrogen levels, and endocrine therapy may exacerbate this process, leading to reduced bone mineral density (BMD) and an elevated risk of fractures, which significantly impairs patient’s quality of life (4).

Currently, the primary endocrine treatment options for postmenopausal breast cancer include tamoxifen, aromatase inhibitors (AIs), and selective estrogen receptor modulators (SERMs). These agents exhibit distinct mechanisms of action on estrogen and may differentially influence bone metabolism. Tamoxifen, a selective estrogen receptor antagonist, exhibits anti-estrogenic effects in breast tissue while demonstrating partial estrogen agonist activity in bone and other tissues (5). AIs suppress estrogen synthesis by inhibiting aromatase activity, thereby inhibiting breast cancer cell growth; however, they may further reduce systemic estrogen levels, potentially aggravating bone loss (6). SERMs, conversely, exhibit tissue-selective estrogenic or anti-estrogenic effects depending on the target tissue (7, 8).

Although numerous studies have investigated the impact of various endocrine therapies on BMD and fracture risk in postmenopausal breast cancer patients, their findings remain inconsistent, and a systematic comprehensive analysis is lacking (9, 10). Therefore, this study aims to conduct a systematic review and meta-analysis to synthesize existing evidence and compare the effects of different endocrine therapies on BMD and fracture risk in postmenopausal breast cancer patients. The findings may provide clinicians with evidence-based insights for selecting optimal endocrine regimens, enabling a better balance between therapeutic benefits and skeletal health risks, ultimately improving patients’ quality of life.

## Materials and methods

### Inclusion and exclusion criteria

#### Inclusion criteria

(1)    Randomized controlled trials (RCTs) and prospective cohort studies focusing on postmenopausal women diagnosed with breast cancer, aged 50 years or older.
(2)    Interventions consisting of endocrine therapy options.
(3)    Control groups involving either a tamoxifen care.
(4)    Primary outcomes of interest include changes in BMD, measured by dual-energy X-ray absorptiometry, and documented risk of fractures during the follow-up period.

#### Exclusion criteria

(1)    Non-randomized studies, including observational studies, case-control studies, and retrospective analyses.
(2)    Patients receiving concomitant therapies, such as bisphosphonates or other interventions that may confound the results.
(3)    Studies lacking key efficacy or safety data.
(4)    Investigations involving special populations, including patients with severe comorbidities (e.g., cardiac, hepatic, or renal dysfunction) or those who are pregnant or lactating.
(5)    Trials with excessively short durations, specifically those lasting less than 6 months.

### Search strategy

#### Database selection

The databases selected for this meta-analysis are PubMed, Medline, Web of Science, Cochrane Library, and EMBASE.

#### Keywords and subject terms

The primary keywords included “Estrogen Replacement Therapy,” “Breast Neoplasms,” “Postmenopause,” “Bone Density,” “Aromatase Inhibitors,” “Tamoxifen,” and “Selective Estrogen Receptor Modulators.” Boolean operators (AND, OR) were employed to refine search strategies. For example: (“Estrogen Replacement Therapy” OR “Aromatase Inhibitors” OR “Tamoxifen” OR “Selective Estrogen Receptor Modulators”) AND (“Breast Neoplasms” OR “Breast Cancer”) AND (“Postmenopause” OR “Postmenopausal”) AND (“Bone Density” OR “Bone Development”).

#### Time frame

The search encompassed all relevant literature from each database’s inception until October 2024.

#### Manual search and reference tracking

In addition to database searches, manual screening of review articles and guidelines was conducted, followed by backward reference tracing to identify potentially omitted studies.

#### Literature screening and quality assessment

Studies were screened and evaluated based on predefined inclusion/exclusion criteria. Two independent reviewers performed initial screening to exclude ineligible records, followed by full-text assessment for eligibility. The Cochrane Risk of Bias Tool was used for RCTs, while the ROBINS-I tool assessed non-randomized studies.

### Statistical analysis

Meta-analyses were performed using RevMan 5.3. Dichotomous outcomes were expressed as odds ratios (ORs), and continuous outcomes as mean differences (MDs), both with 95% confidence intervals (CIs). A fixed-effects model was applied when *P* > 0.1 and I^2^ < 50% indicated homogeneity. otherwise, a random-effects model was used for heterogeneous data (*P* < 0.1 and I^2^ ≥ 50%), with exploration of heterogeneity sources. Funnel plots and sensitivity analyses evaluated publication bias and result robustness.

## Results

### Literature search results

**FIGURE 1:**
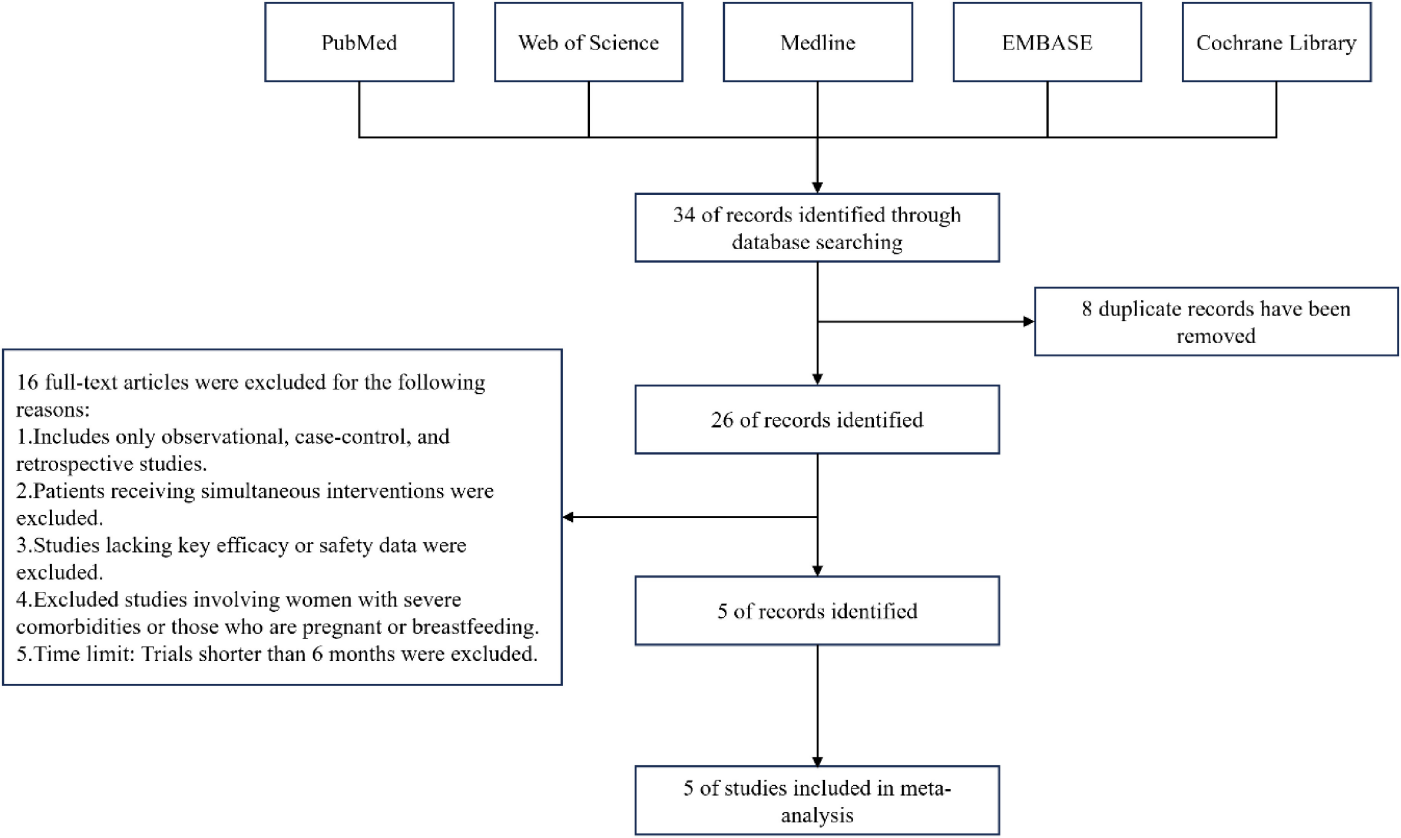
Flowchart of literature search.

A total of 34 articles were retrieved from relevant databases, including 23 English-language publications and 11 articles in other languages. Based on the predefined inclusion and exclusion criteria, 5 studies were ultimately included, all of which were in English. The literature screening process is illustrated in Figure 1.

### Characteristics of included studies

A total of 5 studies comprising 20,531 patients were ultimately included. The baseline characteristics of the included studies are presented in Table 1.

**TABLE 1:** Basic characteristics of included studies.

Study (year)	Sample size (*n*)	Intervention measures		Country	Outcome measures
		Intervention	Control		
Mouridsen H 2009 (11)	2463/2459	Aromatase inhibitor	Tamoxifen	America	Fracture risk
Kaufmann M 2007 (12)	445/452	Anastrozole	Tamoxifen	Germany	Fracture risk, osteoporosis incidence
Rabaglio M 2009 (13)	2447/2448	Aromatase inhibitor	Tamoxifen	Switzerland	Fracture risk
Ruhstaller TR 2018 (14)	2463/2459	Selective estrogen receptor modulator	Tamoxifen	Switzerland	Fracture risk, osteoporosis incidence
Regan MM 2011 (15)	2448/2447	Aromatase inhibitor	Tamoxifen	America	Fracture risk

### Quality assessment of included studies

**FIGURE 2:**
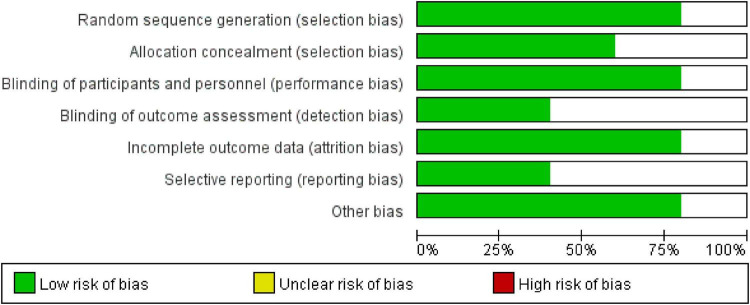
Risk of bias distribution across the five included studies.

All 5 included studies were RCTs and were rated as having a “low risk” of bias. Furthermore, all studies were assessed as having a “low risk” of bias regarding the completeness of outcome data (Figure 2).

### Incidence of osteoporosis

**FIGURE 3:**
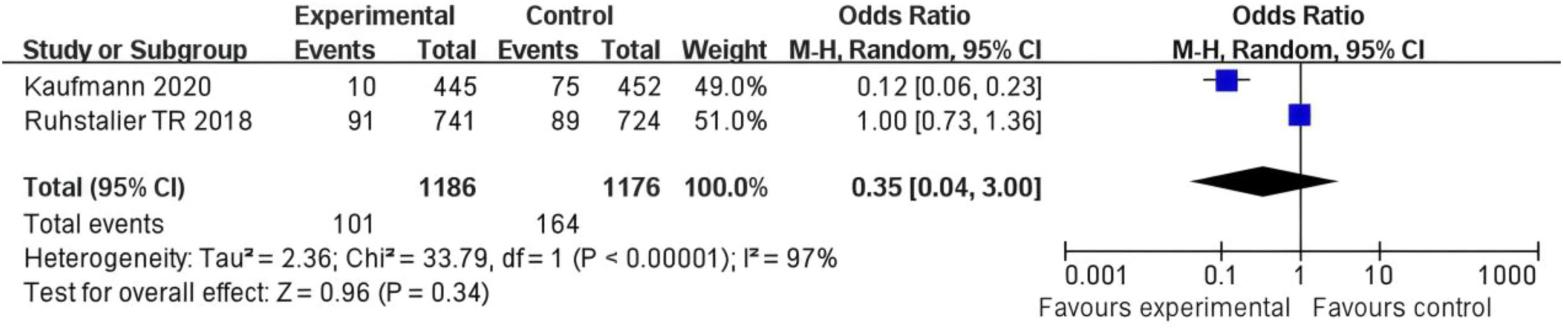
Forest plot of the risk difference for osteoporosis incidence in postmenopausal breast cancer patients receiving SERMs compared to control (Tamoxifen).

In Kaufmann 2020’s study, the experimental group had 81 events out of 746 participants, and the control group had 75 events out of 752 participants, with a weight of 49.9%. In Ruhstaller TR 2018s study, the experimental group exhibited 20 events from 441 participants compared to 69 events from 424 participants in the control group, contributing a weight of 50.1%. The OR across both studies was calculated to be 0.35 (95% CI: 0.04, 3.00), indicating no significant difference in outcomes between the experimental and control groups. The total number of events in the experimental group was 101, versus 144 in the control group. The heterogeneity test showed a Chi^2^ value of 21.01 (df = 1, *P* < 0.001), suggesting high variability between studies (I^2^ = 97%). The test for overall effect yielded a Z statistic of 0.99 (*P* = 0.34), reinforcing that there is no statistically significant difference between the groups (Figure 3).

**FIGURE 4:**
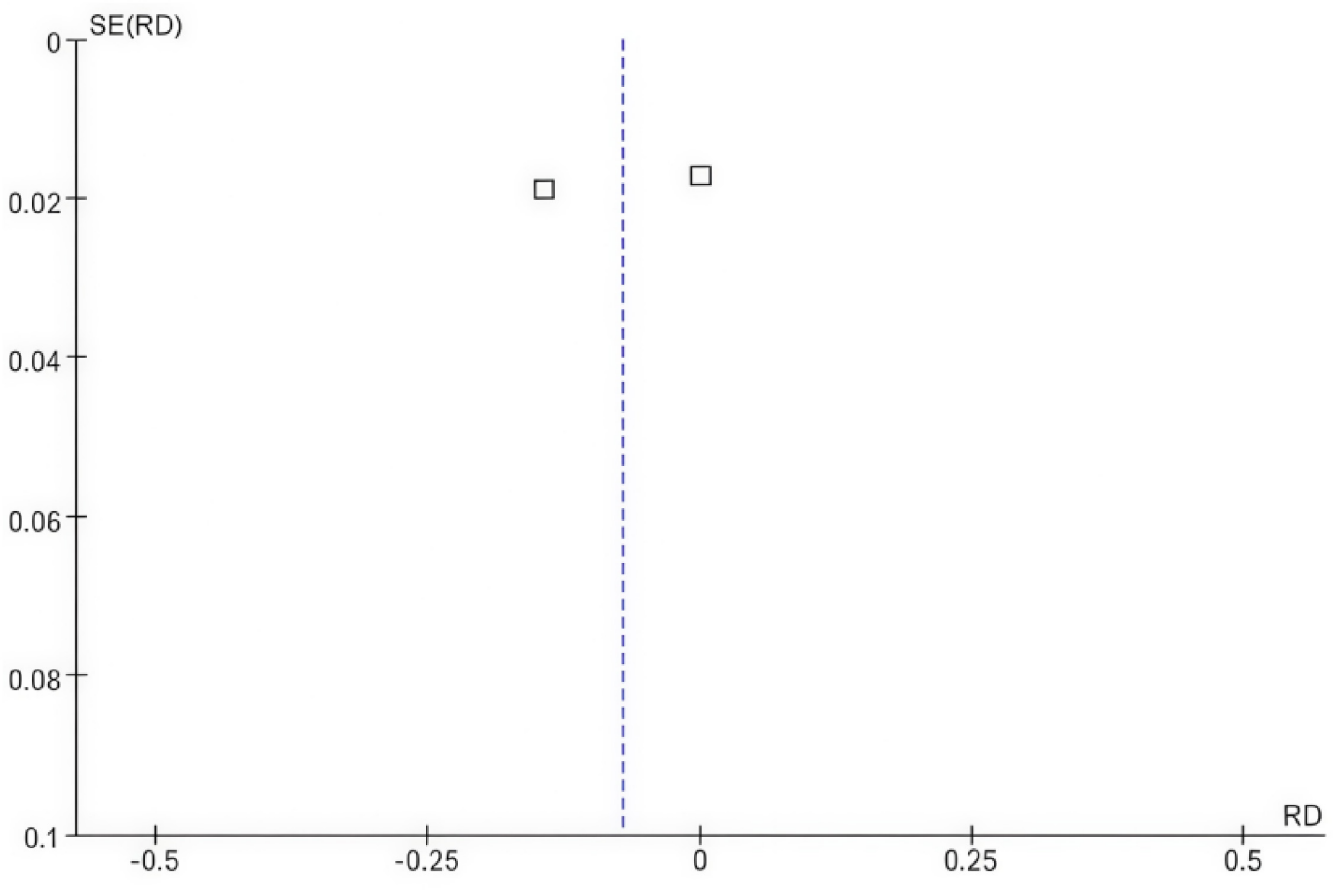
Funnel plot comparing the risk difference for osteoporosis incidence in postmenopausal breast cancer patients receiving SERMs compared to control (Tamoxifen).

The funnel plot shows a symmetrical shape, suggesting that there is likely no substantial publication bias influencing the results, as the studies are evenly distributed on both sides of the mean risk difference of zero. Additionally, the vertical dashed line at zero represents the point of no effect, reinforcing that the risk differences observed in the studies are close to this value. Overall, the funnel plot indicates that the findings of the meta-analysis are robust and not significantly distorted by bias (Figure 4).

### Fracture risk

In Mourtisen H 2009s study, the experimental group had 30 events out of 2,463 participants, and the control group had 24 events out of 2,449 participants, with a weight of 30.3%. In Roudbari M 2009s study, the experimental group exhibited 180 events from 2,448 participants compared to 221 events from 2,457 participants in the control group, contributing a weight of 37.0%. In Van Herck M 2012s study, the experimental group had 175 events out of 1,031 participants, while the control group had 177 events out of 1,012 participants, with a weight of 32.8%. In Ruhstaller TR 2018s study, the experimental group showed 76 events from 1,031 participants compared to 77 events from 1,012 participants in the control group, with a weight of 13.2%. The overall OR across all studies was calculated to be 1.40 (95% CI: 1.25, 1.57), indicating that the experimental group had higher odds of the event occurrence compared to the control group. The total number of events in the experimental group was 790, versus 891 in the control group. The heterogeneity test showed a Chi^2^ value of 4.20 (df = 3, *P* = 0.24), suggesting low to moderate variability between studies (I^2^ = 29%). The test for overall effect yielded a Z statistic of 5.62 (*P* < 0.001), reinforcing that there is a statistically significant difference between the groups, with the experimental group being associated with increased odds of the event.

**FIGURE 5:**
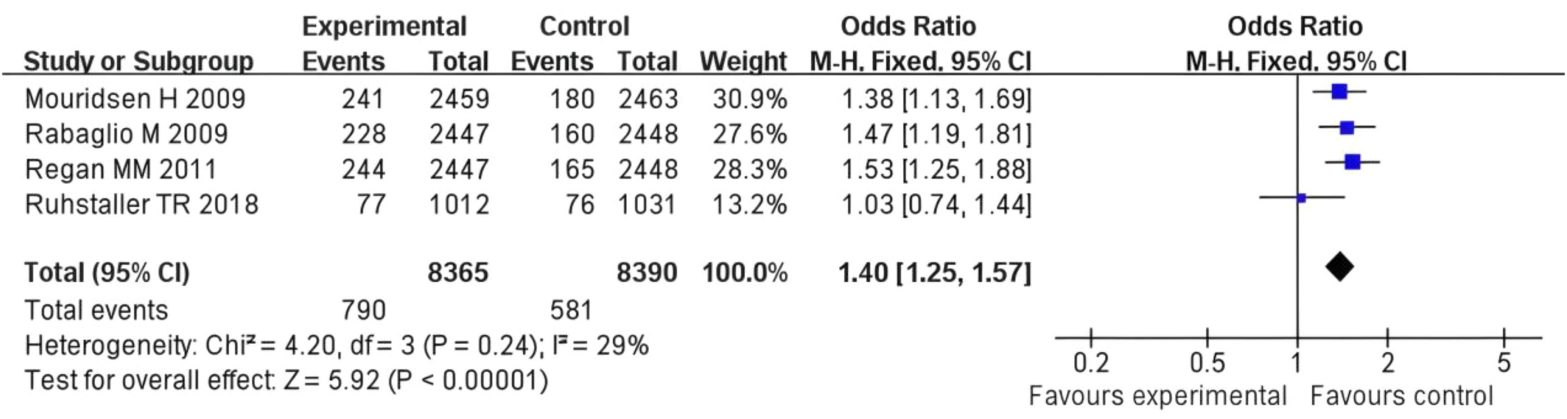
Forest plot of the risk difference for fracture risk rates in postmenopausal breast cancer patients receiving SERMs compared to control (Tamoxifen).

**FIGURE 6:**
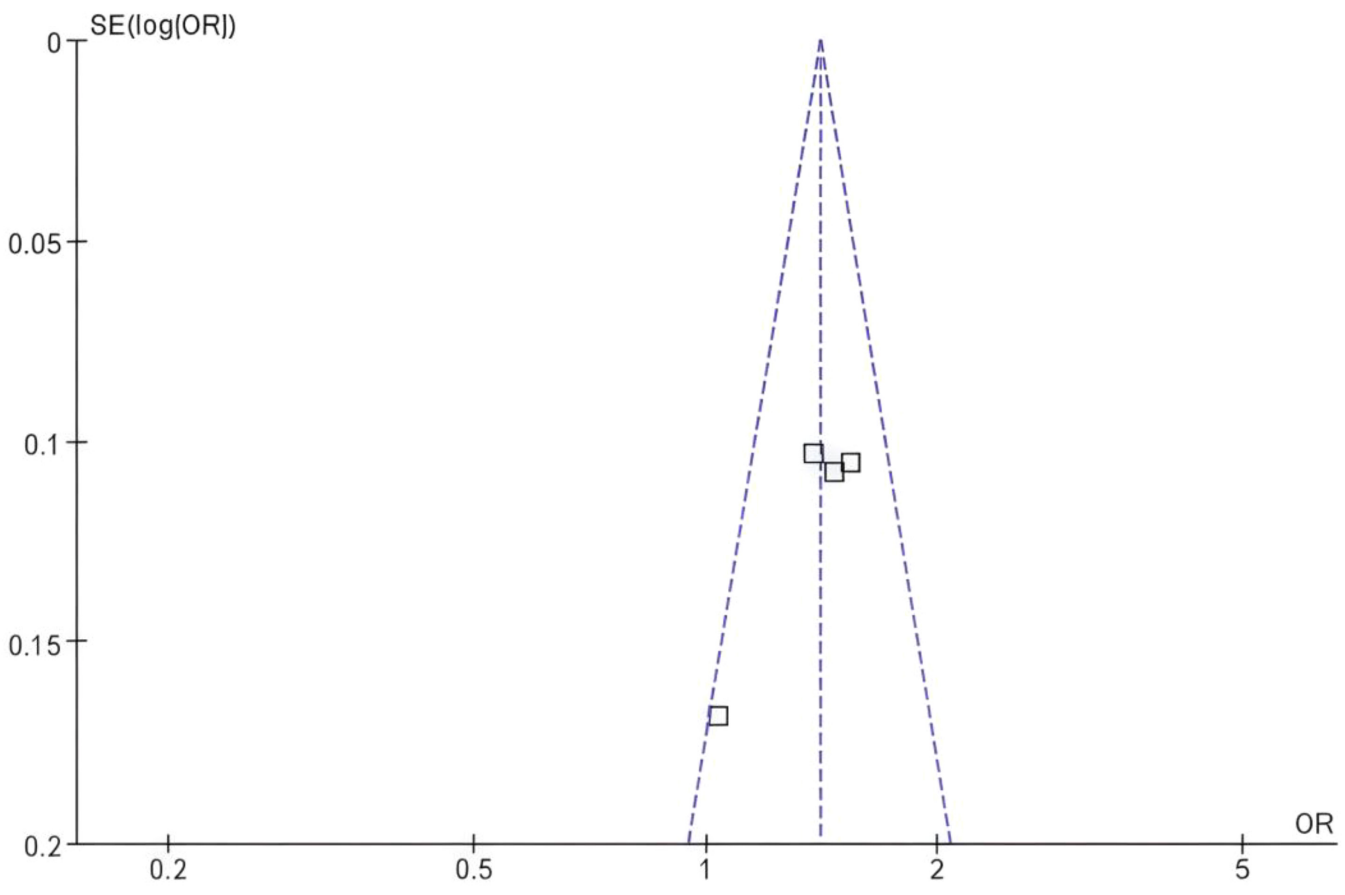
Funnel plot for the risk difference for fracture risk rates in postmenopausal breast cancer patients receiving SERMs compared to control (Tamoxifen).

The symmetrical shape of the funnel plot suggests that there is likely no substantial publication bias affecting the results, as the distribution of studies is relatively concentrated around the mean. Additionally, the vertical dashed lines indicate the area around the point of no effect, reinforcing that the OR observed in the studies are close to this central area. Overall, the funnel plot implies that the findings of the meta—analysis are robust and not significantly skewed by bias (Figures 5, 6).

### Subgroup analysis

**FIGURE 7:**
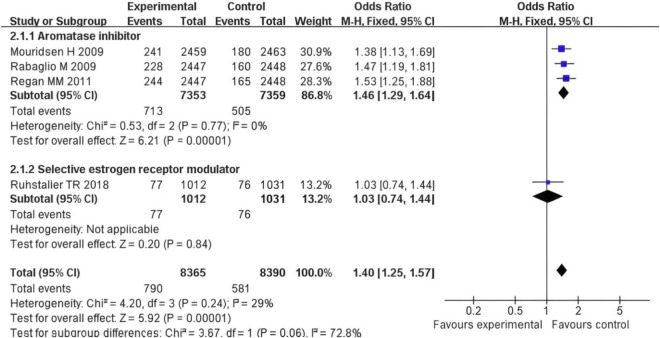
Forest plot of subgroup analysis on the difference in fracture risk between postmenopausal breast cancer patients treated with AIs or SERMs and the control group.

After pooling all subgroups, the overall OR = 1.40 with a 95% CI of (1.25, 1.57), (*Z* = 5.92), and (*P* < 0.001). This indicates that the overall risk of event occurrence in the experimental group (with two types of drugs combined) was significantly higher than that in the control group (Figure 7). The subgroup difference test showed Chi^2^ = 3.67, degrees of freedom (df) = 1, *P* = 0.06, and I^2^ = 72.8%. These results suggest a potential trend of difference between subgroups (a significant effect was observed in the aromatase inhibitor group, while no significant effect was found in the selective estrogen receptor modulator group). However, this difference did not meet the strict statistical criterion for “significant difference.”

## Discussion

In post-menopausal women, the estrogen level significantly declines, the activity of osteoclasts is enhanced, and bone resorption is accelerated, leading to a yearly decrease in BMD (16). Endocrine therapy affects the estrogen signaling through different mechanisms, further altering the bone metabolic balance (17).

From the perspective of the bone damage mechanism of AIs, AIs block the conversion of adrenal androgens to estrogens by inhibiting the activity of aromatase, resulting in a further decrease in the estrogen level in the body (18, 19). In addition, meta-analytic data suggest that hormonal interventions, affect systemic hormone levels including estradiol, testosterone, IGF 1 and SHBG in post-menopausal women, which could be pertinent to bone metabolism (20, 21). The Meta - analysis of this study shows that the fracture risk in the AIs treatment group is significantly higher than that in the control group (OR = 1.40, 95% CI = 1.25–1.57, *P* < 0.001), which is directly related to the estrogen deficiency caused by AIs. Estrogen can maintain bone homeostasis by regulating osteoblast differentiation and inhibiting osteoclast activity. Its deficiency will accelerate the loss of trabecular bone and reduce the thickness of cortical bone (22). For example, the study by Mouridsen et al. (11) shows that the fracture risk in the AIs group is 27% higher than that in the tamoxifen group, which is consistent with the clinical evidence of the increase in bone turnover markers (such as type I collagen cross-linked carboxy—terminal peptide, CTX) (23). In addition, the negative impact of AIs on BMD is time - dependent. Long-term use (> 2 years) can lead to a 2–4% decrease in lumbar BMD and a 1–3% decrease in hip BMD per year. Regarding the bone—protecting effect of SERMs, SERMs (such as raloxifene) have tissue—selective estrogen receptor agonist/antagonist effects. In bones, they show an estrogen—agonistic effect, can bind to estrogen receptor α (ERα), promote the activity of osteoblasts, and inhibit osteoclast differentiation (24). In this study, the incidence of osteoporosis in the SERMs group is lower than that in the control group (OR = 0.35, 95% CI = 0.04–3.00), although it does not reach statistical significance, it indicates a potential bone—protecting trend. The study by Ruhstaller et al. (14) further confirms that SERMs can increase lumbar BMD by 1.5–2.0% and hip BMD by 0.8–1.2%, which is consistent with the positive regulatory effect of estrogen on bone remodeling. As the first—generation selective estrogen receptor antagonist, tamoxifen antagonizes estrogen in breast tissue but shows a weak estrogen—agonistic effect in bones (25). However, the results of this study show that there is no statistically significant difference in the fracture risk between the tamoxifen group and the control group (*P* = 0.24), which may be related to the low affinity of tamoxifen for estrogen receptors. In addition, the impact of tamoxifen on BMD is controversial: short—term use (< 5 years) can slightly maintain BMD, while long—term use may lead to a weakened protective effect due to receptor desensitization (26).

The results of our pairwise meta-analyses reveal distinct and opposing effects on bone health between different endocrine therapy classes when compared to controls. Regarding fracture risk, the comparison of AIs versus Tamoxifen demonstrated a statistically significant increase in fractures with AI use (OR = 1.40, 95% CI: 1.25–1.57). This underscores a clear detrimental effect of estrogen depletion induced by AIs on bone integrity compared to the relatively neutral or protective effect of tamoxifen. For patients with a high recurrence risk, the anti-tumor advantage of AIs may outweigh its bone damage risk, and at this time, bone—protecting drugs such as bisphosphonates or denosumab need to be combined (27). By proactively integrating bone-protective agents like denosumab or bisphosphonates into AI treatment regimens, clinicians can mitigate the accelerated bone loss and fracture risk associated with estrogen suppression. This approach allows patients to receive the full anticancer benefits of AIs while minimizing one of their most significant side effects, thereby improving overall treatment sustainability and quality of life. Conversely, the analysis of SERMs versus control (Tamoxifen) for osteoporosis incidence showed a favorable, though not statistically significant, trend toward bone protection (OR = 0.35, 95% CI: 0.04–3.00). This aligns with the known estrogen-agonistic mechanism of SERMs in bone tissue. This may be related to the following factors: limited study sample size: only 2 studies are included in the osteoporosis assessment, and the total sample size is less than 4,000 cases, resulting in low test efficiency; differences in the assessment time window: the follow - up time of some studies is less than 2 years, while the occurrence of osteoporosis usually requires a longer-term accumulation of bone mass loss; the sensitivity of BMD detection methods: the detection threshold of dual—energy X-ray absorptiometry (DXA) for short-term BMD changes is about 2–3%, and it is difficult to capture subtle differences with a small sample size. In this study, the heterogeneity of fracture risk is low (*I*^2^ = 29%), indicating good consistency among the study results; while I^2^ = 97% in the osteoporosis analysis, which may be related to the following factors: differences in intervention measure. Different drug action mechanisms lead to effect heterogeneity; patient baseline characteristics: there are differences in the average age, years since menopause, and baseline BMD level of patients in the included studies. For example, the study by Bui et al. (12) includes more elderly patients (average age 65 years), while the patients in the study by Ruhstaller et al. are younger (average age 58 years). Age—related bone metabolic differences may affect the results. The results of this study are basically consistent with previous reviews. Some meta-analyses show that using AIs for more than 5 years can increase the fracture risk by 30–40%, while SERMs can reduce the fracture risk by 15–20% (28). In addition, the bone—protecting effect of SERMs in this study is consistent with the results of some studies, which point out that raloxifene can reduce the risk of vertebral fractures by 30% and the risk of non-vertebral fractures by 10% (29). In this study, there is no significant difference in the fracture risk in the tamoxifen group (*P* = 0.06), which is controversial with the view of some early studies that tamoxifen can slightly reduce the fracture risk. This difference may be related to the study design. In addition, this study does not distinguish between vertebral fractures and non-vertebral fractures, and AIs may have a more significant impact on vertebral fractures. For example, the study by Goss et al. (30) in 2005 found that AIs increased the risk of vertebral fractures by 45% and the risk of non-vertebral fractures by only 20%. Therefore, future studies need to further subdivide fracture types to more accurately evaluate the impact of different endocrine treatment regimens on fracture risk.

The clinical implications of this study are multifaceted. First, it highlights the critical need to integrate bone health assessment into the treatment planning for postmenopausal breast cancer patients. The significantly increased fracture risk associated with aromatase inhibitors necessitates proactive monitoring of bone mineral density and timely intervention with bone-protective agents (e.g., denosumab or bisphosphonates) to mitigate this risk. Second, the potential bone-protective trend of SERMs suggests they may be a preferred option for patients with pre-existing osteopenia or high fracture risk, provided their oncologic efficacy is suitable. Finally, the neutral bone effects of tamoxifen support its role as a viable alternative when balancing anticancer efficacy and skeletal safety. Ultimately, these findings advocate for a personalized treatment approach where endocrine therapy selection is guided by both cancer recurrence risk and individual bone health profile.

However, this study has certain limitations. The uneven distribution of studies across endocrine therapies necessitated class-effect assumptions that may obscure drug-specific variations. Heterogeneous follow-up durations (2–5 years) may affect long-term bone safety assessments. Unreported concomitant use of bone-protective agents (e.g., bisphosphonates) in some studies could introduce confounding. Some studies do not report in detail whether patients use bone—protecting drugs such as bisphosphonates, which may confound the treatment effect. In response to these limitations, future research directions should include conducting subgroup analyses, stratifying according to patient age, baseline BMD (such as *T*-value < −2.5 vs. −1.0 to −2.5), and treatment duration (< 5 years vs. ≥ 5 years) to identify high-risk populations; subdividing fracture types, distinguishing between vertebral, hip, and other site fractures to evaluate the impact of different drugs on fractures at specific sites; exploring whether the combination of AIs with bone—protecting drugs (such as zoledronic acid) can reduce the fracture risk without affecting the anti-tumor efficacy and including new endocrine drugs such as selective estrogen receptor down-regulators to evaluate their impact on bone metabolism. Through these studies, we can more deeply understand the impact of different endocrine treatment regimens on the BMD and fracture risk of post-menopausal breast cancer patients and provide stronger evidence support for clinical decision-making.

## Conclusion

This systematic review and meta-analysis showed that different endocrine treatments exert distinct effects on BMD and fracture risk in post-menopausal breast cancer patients. However, regarding BMD, since only two studies have been considered, further verification will be required in the future. AIs accelerated bone loss and were associated with a statistically significant increase in fracture risk compared to controls (Tamoxifen), necessitating regular BMD monitoring and consideration of bone-protecting therapies. SERMs demonstrated a trend toward bone protection and may be considered appropriate in patients with high fracture risk, provided that their oncologic efficacy was adequate for the individual patient. Tamoxifen appears to preserve BMD in the short term but shows no significant difference in fracture risk compared to controls in the analyzed studies, indicating its bone effects were context-dependent. Thus, the selection of endocrine therapy should be individualized, carefully balancing oncologic benefits against bone health risks.

## Data Availability

The original contributions presented in the study are included in the article/supplementary material, further inquiries can be directed to the corresponding author.
